# HIPK2 is a new drug target for anti-fibrosis therapy in kidney disease

**DOI:** 10.3389/fphys.2015.00132

**Published:** 2015-04-28

**Authors:** Melinda M. Nugent, Kyung Lee, John Cijiang He

**Affiliations:** ^1^Department of Medicine/Nephrology, Icahn School of Medicine at Mount SinaiNew York, NY, USA; ^2^Renal Section, James J. Peter Veterans Administration Medical CenterNew York, NY, USA

**Keywords:** kidney, fibrosis, HIPK2, inflammation, signaling pathways

## Abstract

*In vitro* and animal studies continue to elucidate the mechanisms of fibrosis and have led to advancements in treatment for idiopathic pulmonary fibrosis and cirrhosis, but the search for treatments for renal fibrosis has been more disappointing. Here, we will discuss homeodomain-interacting-protein kinase 2 (HIPK2), a novel regulator of fibrosis that acts upstream of major fibrosis signaling pathways. Its key role in renal fibrosis has been validated *in vitro* and in several murine models of chronic kidney diseases (CKD).

## Background: fibrosis and ESRD

Fibrosis is the final common pathway for chronic kidney diseases (CKD). Renal fibrosis occurs as an overwhelming response to tissue injury. The attempt to repair damage begins with the recruitment of inflammatory cells, but ends with an unchecked inflammatory response that activates matrix-producing cells leading to tubular cell apoptosis, irreversible scarring, loss of renal function, and ultimately end stage renal disease (ESRD) (Chuang et al., 2012). CKD and ESRD are major public health problems with increasing prevalence worldwide and are associated with early mortality, poor quality of life, and high healthcare costs. Unfortunately, at present, there are no effective therapies to prevent or slow the progression of renal fibrosis, and the treatment options for patients with ESRD are limited to dialysis and renal transplant. The blockade of the renin–angiotensin–aldosterone system (RAAS) that nephrologists currently rely on for renal protection decreases the risk of progression by only 20% (Drawz and Rosenberg, 2013). To develop effective molecular targets for prevention and treatment of renal fibrosis, it is important to gain a better understanding of the mechanisms involved.

Here, we will review major signaling pathways that contribute to aberrant wound healing and fibrosis with a focus on homeodomain-interacting-protein kinase 2 (HIPK2) which acts upstream of several pro-fibrosis pathways.

## Signaling pathways in renal fibrosis and targeted therapies

Novel drugs targeting the signaling pathways of renal fibrosis have generally given mixed results and occasionally concerning side effects. The shortcomings of these experimental therapies are likely due to the fact that the pathogenesis of fibrosis is complex; blockade of one pathway may still leave open many others for promoting inflammation and fibrosis.

A central mediator in renal fibrosis is transforming growth factor-β (TGF-β). TGF-β superfamily is highly conserved and plays a major role in a variety of cellular responses including cell proliferation, differentiation and apoptosis. TGF-β1, the predominant isoform, stimulates extracellular matrix production via Smad-dependent and Smad-independent mechanisms to inhibit extracellular matrix degradation and promote expression of pro-fibrosis genes in various cell types (Blobe et al., 2000; Massagué and Wotton, 2000). TGF-β has also been shown to exert pleiotropic effects in various cell types. In focal segmental glomerulosclerosis, hyperplastic podocytes exhibit increased TGF-β/Smad signaling which results in mesangial cell matrix overproduction (Kim et al., 2003). In animal models of diabetic nephropathy, increased TGF-β1 signaling leads to podocyte apoptosis and glomerulosclerosis (Kim et al., 2003).

Recently, Eli Lilly sponsored a phase 2 trial for a humanized neutralizing monoclonal antibody against TGF-β1 for the treatment of advanced diabetic nephropathy, but the study had to be prematurely terminated due to futility in efficacy. These data were presented at the ASN 2014 and several issues of the study design were raised by experts in the field during the meeting. There are also concerns regarding anti-TGF-β therapies, since in addition to its pro-fibrotic effects, it also exerts anti-inflammatory effects; indiscriminate complete blockade of TGF-β functions can promote inflammation.

Despite some disappointments in the field of renal fibrosis, the FDA has recently approved two promising anti-fibrosis drugs, pirfenidone and nintedanib, for the treatment of idiopathic pulmonary fibrosis (IPF). These drugs are appealing because they block chronic inflammation in addition to decreasing aberrant wound healing. Pirfenidone has well-established anti-fibrotic and anti-inflammatory properties in various *in-vitro* systems and animal models of fibrosis (Schaefer et al., 2011). A number of cell-based studies have shown that pirfenidone reduces fibroblast proliferation, inhibits TGF-β stimulated collagen production and reduces the production of fibrogenic mediators such as TGF-β (Lee et al., 1998; Nakayama et al., 2008; Lin et al., 2009). Pirfenidone has also been shown to reduce production of inflammatory mediators such as TNF-α and IL-1β (Phillips et al., 2005). These activities are consistent with the broader anti-fibrotic and anti-inflammatory activities observed in animal models of fibrosis. Pirfenidone is a drug developed by several companies worldwide for the treatment of IPF. In 2008, it was first approved in Japan for the treatment of IPF. While pirfenidone has established safety and efficacy in treating human pulmonary fibrosis and cirrhosis and is able to prevent kidney fibrosis in rodents, these benefits have not yet been confirmed in renal patients. In a study with 77 patients with kidney disease, pirfenidone improved renal function but failed to significantly reduce proteinuria (Ramachandrarao et al., 2009), suggesting that pirfenidone improves renal fibrosis but does not improve podocyte injury. Nintedanib is a small molecule tyrosine-kinase inhibitor targeting vascular endothelial growth factor, fibroblast growth factor receptor and platelet derived growth factor receptor as an anti-angiogenesis and anti-cancer agent (Roth et al., 2015). The role of Nintedanib has never been determined in kidney disease.

Tranilast is another agent that is an anti-allergic drug and has been used for the treatment of allergic disorders such as asthma, allergic rhinitis and atopic dermatitis. However, *in-vitro* studies show that it reduces collagen synthesis in fibroblasts, and inhibits the production of interleukin-6 in endothelial cells (Spiecker et al., 2002). Tranilast has been shown to improve renal function in the subtotal nephrectomy CKD model in rodents and to reduce albuminuria and slow progression of kidney disease in a small cohort of renal patients (Soma et al., 2002; Spiecker et al., 2002). However, when tranilast was used in patients with cardiovascular disease to prevent major cardiovascular events and restenosis after percutaneous coronary intervention, there was a rise in creatinine afterwards suggesting that tranilast has some nephrotoxicity (Holmes et al., 2002).

Bone-morphogenetic protein-7 (BMP-7) is part of the TGF-β family and actually opposes the pro-fibrogenic effects of TGF-β1 by blocking transcriptional upregulation of plasminogen activator inhibitor-1 and antagonizing TGF-β-induced epithelial-mesenchymal transition (Zeisberg et al., 2003). BMP-7 expression is reduced in both animal models of diabetic nephropathy (DN) and in DN patients, and is also downregulated in the rodent model of ischemic acute tubular necrosis (Vukicevic et al., 1998; Simon et al., 1999; De Petris et al., 2007). Consistent with its TGF-β antagonizing role, exogenous recombinant BMP-7 administration *in vivo* led to significant reduction of renal fibrosis in pre-clinical models of diabetic nephropathy and CKD (Wang, 2006). There are currently several phase 2 clinical trials of agonists of BMP-7 signaling underway.

Connective tissue growth factor (CTGF) modulates TGF-β and BMP-7 activities. CTGF is not expressed in normal kidney but is upregulated in human kidney disease where it enhances TGFβ-1 signaling resulting in myofibroblast activation, fibronectin accumulation and tissue fibrosis (Yokoi et al., 2001; Guha et al., 2007; Wang et al., 2011). In mouse models of kidney disease, the reduction of CTGF prevents renal fibrosis. In a small clinical trial, human anti-CTGF monoclonal antibody significantly reduced albuminuria in patients with diabetic nephropathy without significant adverse events (Adler et al., 2010). FibroGen, Inc. started a phase 2 clinical trial designed to evaluate the efficacy and safety of FG-3019, a fully human monoclonal antibody against CTGF in patients with type 2 diabetes and advanced kidney disease in 2009. Unfortunately, this clinical trial was terminated because of some regulation issues.

Despite some promising findings in animal studies and small clinical trials, currently, anti-fibrosis drugs have yet to be effectively translated into clinical practice in the field of nephrology. The current strongest tool for renal protection remains to be RAAS blockade which decreases the pro-fibrotic effects of angiotensin II, thereby decreasing TGF-β and TGF-β receptor levels in the kidney and decreasing expression of CTGF. However, RAAS blockade is insufficient against renal fibrosis as it only partially decreases the risk of progression, which highlights the urgency for the development of more effective treatments for renal fibrosis.

## HIPK2 in renal fibrosis

In order to determine which signaling networks are critical in renal fibrosis Jin et al. developed a combined systems biology and experimental approach to identify major signaling pathways involved in gene expression changes in renal fibrosis (Jin et al., 2012; Chen et al., 2013). TG-26 mice were used because they are the murine model of HIV-associated nephropathy and are a model of CKD. Their pathology is characterized by diffuse segmental and global glomerulosclerosis with podocyte foot process effacement, as well as significant tubulointerstitial injury accompanied by heavy proteinuria, elevated blood urea nitrogen, edema and hypoalbuminemia (Rosenstiel et al., 2009). Jin et al. found differentially regulated transcription factors in the kidneys of Tg-26 mice compared to wild-type control mice and then identified upstream protein kinases that are likely to phosphorylate those transcription factors. HIPK2 was identified as one of the protein kinases with the highest ranks using this approach. Subsequent experiments using gene ablation of HIPK2 showed that it is a master regulator of kidney fibrosis in experimental models of CKD.

## HIPK2 structure and function

**Figure 1:**
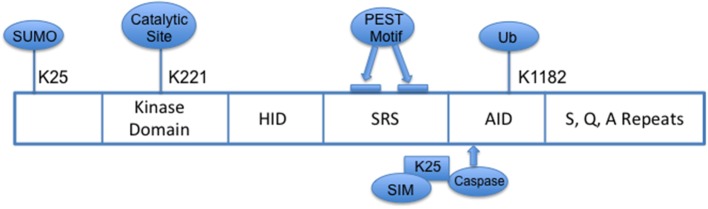
**Schema of HIPK2 Structure**. HID, Homeoprotein-interacting domain; SRS, Speckle-retention signal; AID, auto-inhibitory domain; PEST, peptide sequence rich in proline (P), glutamic acid (E), serine (S) and threonine (T); SIM, SUMO-interacting motif; Ub, ubiquitylation.

HIPK2 is part of the family of conserved serine/threonine homeodomain-interacting kinases that localizes to nuclear bodies. Structurally, its N-terminal domain consists of a sumoylation site at lysine 25 and a 330 amino acid serine/threonine kinase domain with a key catalytic site at lysine residue, K221 (Figure 1) (Gresko et al., 2005; Hofmann et al., 2005); the homeobox-interacting domain (HID), a protein–protein interaction region, is followed by the speckle-retention signal (SRS) which contains two PEST motifs and is required for the subcellular localization of HIPK2 to nuclear bodies (Kim et al., 1998); and its C-terminus contains the auto-inhibitory domain (AID) with a ubiquitination site (K1182), which, when cleaved by caspases leads to full activation of HIPK2. HIPK2's pleotropic functions include serving as co-regulator of transcription factors, modulator of growth, development, morphogenesis and cell death, tumor suppressor, and regulator of response to DNA damage (Hofmann et al., 2001; D'Orazi et al., 2002; Link et al., 2007; Bitomsky and Hofmann, 2009; Calzado et al., 2014). A defective HIPK2-activated P53 system has been implicated in the pathogenesis of idiopathic pulmonary fibrosis (Ricci et al., 2013).

In renal tubular epithelial cells (RTEC), HIPK2 induced expression of pro-fibrosis markers and apoptosis in HIV-infected RTEC, likely due to its role in activating p53, TGF-β, and Wnt/Notch pathways, which are each known to increase expression of pro-fibrosis markers (Puca et al., 2010). Conversely, suppression of HIPK2 activity via kinase dead (KD)-HIPK2 expression prevented TGF-β-induced expression of fibrosis markers and attenuated HIV-induced apoptosis. In addition, overexpression of WT-HIPK2 in HIV-infected RTECs enhanced the expression of NF-κB targeted genes, while overexpression of KD-HIPK2 suppressed NF-κB activation and inhibited the expression of its downstream target genes. These data suggest that HIPK2 mediates not only the fibrosis pathway but also the inflammatory pathway. These results were further validated *in vivo* using three different murine models of CKD (unilateral ureteral obstruction or UUO, folic acid nephropathy and HIVAN) by genetic ablation of HIPK2. In all three separate models of CKD, loss of HIPK2 resulted in attenuation of renal fibrosis, consistent with the role of HIPK2 as a regulator of gene expressions involved in tubular injury and fibrosis.

## HIPK2 regulation is at post-translational level

HIPK2 is upregulated in fibrosis in murine kidney disease and in human HIVAN, focal segmental glomerulosclerosis, diabetic nephropathy and IgA nephropathy. However, this upregulation appeared to be mediated through a post-translational regulation, as the mRNA levels did not differ in Tg26 kidneys, while there was a significant increase in protein detected by immunohistochemistry. Indeed, the HIPK2 level was found to be regulated through seven in absentia homolog-1 (SIAH-1) mediated proteasomal degradation. However, oxidative stress, such as from HIV infection, decreased SIAH-1 mRNA and protein expression *in vitro*, resulting in an accumulation of HIPK2. This inverse relationship between SIAH-1 and HIPK2 expression was confirmed *in vivo* in Tg26 mice, which had decreased SIAH-1 expression in the renal cortex. While these findings are intriguing, further reduction of HIPK2 staining in late HIVAN glomeruli did not correlate with SIAH-1 expression suggesting that there are other mechanisms besides SIAH-1 that regulate HIPK2. In fact, studies suggest that HIPK2 expression is also downregulated by miR-141 interaction with HIPK2′s 3′ untranslated region which leads to upregulated e-cadherin, downregulated vimentin, and ultimately decreased epithelial to mesenchymal transition (Huang et al., 2015). The role of reactive oxidative species in HIPK2 expression was further confirmed when treatment with n-acetylcysteine, a reactive oxidative species scavenger, attenuated the HIV-induced increase in caspase 3 activity and epithelial-mesenchymal transition marker expression in RTECs *in vitro* (Jin et al., 2012) and resulted in increased SIAH1 expression and reduced HIPK2 expression in Tg26 mice, ultimately resulting in a partial reduction in proteinuria, and attenuation of azotemia and kidney fibrosis.

## Pros and cons of HIPK2 as potential anti-fibrosis target

The above data suggest that inhibition of HIPK2 would mitigate renal fibrosis progression in kidney disease. Importantly, genetic ablation of HIPK2 does not result in a significant phenotype, as opposed to the detrimental effects seen with partial NF-κB inhibition, making HIPK2 an attractive target for therapy (Doi et al., 1997). Although recent studies suggest that it is possible to develop specific HIPK2 inhibitors (Cozza et al., 2014), at present, there are no specific HIPK2 inhibitors available for clinical testing. Development of specific HIPK2 inhibitors is necessary, since unlike the HIPK2 KO mice that show no overt phenotype, HIPK1 and HIPK2 double KO mice are lethal.

One concern of using HIPK2 inhibitors as anti-fibrosis therapy is its tumor suppressive effects. HIPK2 suppresses tumor growth by activating p53 and inducing tumor cell apoptosis (Li et al., 2007; Wei et al., 2007; Stanga et al., 2010; D'Orazi et al., 2012; Mao et al., 2012). In addition to having tumor-suppressive effects, HIPK2 is also needed for TGF-β-mediated survival of midbrain dopaminergic neurons (Zhang et al., 2007; Lanni et al., 2010; Stanga et al., 2010; Chalazonitis et al., 2011). The potential tumor suppressive and neurologic effects of HIPK2 suggest that inhibitors of HIPK2 could have potential adverse effects which need to be carefully watched for in future clinical studies. However, it is likely that the effects of HIPK2 are quite different between tumor cells and non-tumor cells and HIPK2 inhibitors could be used in CKD patients without history of cancers using the same criteria that we currently use for immunosuppressive medication in patients with kidney disease.

## Conclusion

Several key signaling pathways that mediate renal fibrosis have been identified. This has been shown both *in vitro* in cultured kidney cells and *in vivo* in animal models of kidney disease. However, currently, none of the drugs targeting these pathways have been proven to be effective anti-fibrosis therapy for kidney disease in large clinical trials. One potential reason is that renal fibrosis is mediated by a complex signaling network of several different pathways. Therefore, targeting one pathway is not enough to be effective. HIPK2 has been identified as a master regulator of kidney fibrosis and acts upstream of several major fibrosis-signaling pathways. Controlling HIPK2's activities might be an effective approach to reduce the progression of kidney fibrosis. However, it is important to note that HIPK2 also has some onco-suppressor functions and that loss of HIPK2 may also lead to neurodegenerative disease (Lanni et al., 2010). Therefore, we need to carefully monitor for potential side effects during future clinical studies. Further structural and functional studies of HIPK2 may aid in the development of more specific HIPK2 inhibitors as anti-fibrosis therapy without causing these side effects.

### Conflict of interest statement

The authors declare that the research was conducted in the absence of any commercial or financial relationships that could be construed as a potential conflict of interest.

## Abbreviations

HIPK2: homeodomain-interacting-protein kinase 2
RAAS: renin–angiotensin–aldosterone system
TGF-β: transforming growth factor-β
BMP-7: bone-morphogenetic protein-7
CTGF: connective tissue growth factor
RTEC: renal tubular epithelial cells
SIAH-1: seven in absentia homolog-1.
